# Hyper-resting brain entropy within chronic smokers and its moderation by Sex

**DOI:** 10.1038/srep29435

**Published:** 2016-07-05

**Authors:** Zhengjun Li, Zhuo Fang, Nathan Hager, Hengyi Rao, Ze Wang

**Affiliations:** 1 Department of Psychiatry, Perelman School of Medicine, University of Pennsylvania, Philadelphia, Pennsylvania, USA; 2 Laboratory of Applied Brain and Cognitive Sciences, College of International Business, Shanghai International Studies University, Shanghai, China; 3 The Brain and Mind Institute, Department of Psychology, Western University, London, Ontario, Canada; 4 Department of Neurology, Perelman School of Medicine, University of Pennsylvania, Philadelphia, Pennsylvania, USA; 5 Center for Cognition and Brain Disorders, Institutes of Neurological Science, Hangzhou Normal University, Wenzhou RD 126, Building 7, CCBD MR, Hangzhou, Zhejiang Province, 310005, China; 6 Affiliated Hospital of Hangzhou Normal University, Wenzhou RD 126, Building 7, CCBD MR, Hangzhou, Zhejiang Province, 310005, China; 7 Zhejiang Key Laboratory for Research in Assessment of Cognitive Impairments, Hangzhou, Zhejiang Province, China

## Abstract

Cigarette smoking is a chronic relapsing brain disorder, and remains a premier cause of morbidity and mortality. Functional neuroimaging has been used to assess differences in the mean strength of brain activity in smokers’ brains, however less is known about the temporal dynamics within smokers’ brains. Temporal dynamics is a key feature of a dynamic system such as the brain, and may carry information critical to understanding the brain mechanisms underlying cigarette smoking. We measured the temporal dynamics of brain activity using brain entropy (BEN) mapping and compared BEN between chronic non-deprived smokers and non-smoking controls. Because of the known sex differences in neural and behavioral smoking characteristics, comparisons were also made between males and females. Associations between BEN and smoking related clinical measures were assessed in smokers. Our data showed globally higher BEN in chronic smokers compared to controls. The escalated BEN was associated with more years of smoking in the right limbic area and frontal region. Female nonsmokers showed higher BEN than male nonsmokers in prefrontal cortex, insula, and precuneus, but the BEN sex difference in smokers was less pronounced. These findings suggest that BEN mapping may provide a useful tool for probing brain mechanisms related to smoking.

Cigarette smoking is a chronic relapsing disorder and is a leading cause of preventable disease and premature death^1^. As such, understanding the neural mechanisms underlying nicotine addiction represents a high research priority which may impact future treatment strategies. Over the past few decades, functional neuroimaging has been increasingly used to probe smoking-related brain signatures. While the bulk of such studies focus on brain activations in response to smoking cues or during task performance^2^,^3^,^4^,^5^, recent years have seen increased research interest in resting state brain activity which accounts for most of brain energy consumption^6^,^7^.

Most resting state studies in cigarette smoking focused on inter-regional functional connectivity (FC) using the seed-based FC analysis, resting network analysis, or functional connectome (FCM). Using anatomically-defined segments of the cingulate cortex as the seed regions, Hong *et al*.^8^ found that reduced dorsal anterior cingulate cortex (dACC)-striatum FC in 3.5 hours nicotine-deprived smokers correlated with higher nicotine dependence level, while short-term nicotine administration improved the cingulate-neocortical FC. Using the entire hemisphere as the seed region-of-interest, Viswanath and colleagues showed increased inter-hemispheric FC in sated cigarette smokers as compared to controls, which was positively correlated with the number of cigarettes smoked per day^9^. Stoeckel *et al*. reported lower anterior insula FC in smokers than controls^10^. In^11^, the anterior insula was also used as a seed, and the authors reported reduced FC in ACC, dorso-lateral prefrontal cortex (dlPFC), ventro-medial PFC (vmPFC) and striatum. The dACC FC was demonstrated to be associated with risky decision-making in smokers^12^. Using resting network analysis, Janes and colleagues found greater coupling between left fronto-parietal and medial prefrontal cortex (mPFC) in smokers than in controls^13^. Further, the strength of coupling correlated with the smoking-cue induced brain activity in dorsal striatum^13^. Based on FCM analysis, Lin *et al*.^14^ found that as compared to non-smokers, smokers had lower global but higher local brain network communicating efficiency, consistent with the findings in a recent FCM study in a poly-drug (including cigarette smoking) user population^15^.

Since FC is derived from the coupling between two or more brain regions, one complementary and important research interest is to find a direct measure of regional resting brain activity, which can be subsequently used for brain function or brain deficit localization. Thus far to our knowledge, there was only one such study published in the realm of cigarette smoking^16^, which examined the regional resting state activity using the regional homogeneity (ReHo). As compared to controls, smokers showed decreased ReHo in brain regions associated with the default-mode, frontoparietal attention, and inhibitory control networks, but increased ReHo in regions related to motor planning^16^. ReHo was proposed by Zang *et al*.^17^. At each voxel, ReHo characterizes the synchronization (Kendall’s tau coefficient) of its neighboring voxels’ rsfMRI time series. What ReHo refers is actually not the characteristics of the resting activity dynamics at each local voxel but rather an inter-voxel coherence within the neighboring voxels.

A novel technique has recently been evaluated by our group and others, referred to as brain entropy (BEN) mapping, which provides a direct means to quantify the regional resting state brain activity dynamics^18^,^19^,^20^,^21^,^22^,^23^. Entropy is a physical and statistical measure that indicates the irregularity or incoherence, and therefore the status of a dynamic system^24^ such as human brain. Using electrophysiological data, BEN has long been used as a global index for assessing different brain states^25^,^26^,^27^,^28^,^29^. With fMRI, we have recently demonstrated that regional BEN can be reliably mapped in the normal brain^20^,^21^. The normal BEN distributions, however, may be altered in disease conditions, and can be assessed as potential disease biomarkers. Indeed, altered BEN has been demonstrated in aging^30^, schizophrenia^31^, attention deficit hyperactivity disorder^32^, and the relapsing-remitting multiple sclerosis^33^. Drug addiction including nicotine dependence is known as a disorder of altered brain function brought on by psychoactive substances use, and it is possible that such alterations may manifest as altered BEN, but this has not yet been assessed.

Thus, the purpose of this study was to assess resting BEN in cigarette smokers. We compared BEN between chronic smokers and non-smoking controls and examined the relationship between BEN and smoking related behavioral measures. Because sex differences profoundly affect brain and behavioral endpoints in smokers, as well as treatment response and ability to remain abstinent^34^,^35^,^36^,^37^, differences in BEN between female and male chronic smokers were also explored.

## Methods and Materials

### Subjects

This cross-sectional study included 68 chronic cigarette smoking individuals, hereafter referred to as smokers (35 males, age: 35.3 ± 12.6 (mean ± standard deviation (s.d.)), age ranged from 19 to 58), and 66 individuals who have smoked less than 10 cigarettes throughout their lifetime, hereafter referred to as controls (33 males, age 33.0 ± 8.2 (mean ± s.d.), age ranged from 21 to 51). Detailed demographic information is listed in Table 1. The subjects were recruited from the local community in the West Philadelphia area. There was no significant difference between smokers and controls in age (p = 0.21), or sex (χ^2^ = 0.03, p = 0.87), or ethnicity (χ^2^ = 3.99, p = 0.26). All study procedures adhered to the Declaration of Helsinki and were approved by the University of Pennsylvania Institutional Review Board (IRB). All subjects provided written informed IRB-approved consent prior to participating in study procedures.

### Smoking behaviors

The intensity of physical addiction to nicotine was assessed using the Fagerstrom Test for Nicotine Dependence (FTND)^38^, which is a six-item, self-report test evaluating the compulsion to use cigarette, quantity of consumption and the dependence. It scores from 0 to 10, with 10 being the most severe.

Exposure to tobacco smoke was assessed using self-reported quantities of cigarettes per day (CPD), years-smoking, and pack-years. The years-smoking measures the duration of smoking history in the form of integer years. It was calculated by subtracting the age at smoking initiation from the age at assessment. Pack-years summarizes the amount of cigarettes a person has smoked over a lifetime, e.g., 1 pack-years is equal to smoking a pack (20 manufactured cigarettes) per day for one year. So the pack-years was calculated as CPD/20 *years-smoking.

For the current smoker group, the FTND score was 4.7 ± 1.6 (mean ± s.d.), indicating moderate dependence on nicotine. The smokers smoked 14.5 ± 6.1 CPD, and had 15.4 ± 11.5 years-smoking (mean ± s.d.). The accumulated pack-years for the smokers was 11.4 ± 9.8 (mean ± s.d.).

### Imaging parameters

Smokers smoked ad lib throughout the study and smoked a cigarette to satiety approximately 20 minutes prior to scanning sessions in order to dissipate nicotine induced vascular effects^39^. All MR images were acquired on a Siemens 3 Tesla Trio whole-body scanner (Erlangen, Germany) at the Hospital of the University of Pennsylvania using an eight-channel array coil. High-resolution T1-weighted images were obtained using a 3D-MPRAGE sequence (TR = 1620 ms, TE = 3 ms, flip angle = 15°, slice thickness = 1.0 mm). rsfMRI images were acquired using a T2*-weighted gradient echo echo-planar-imaging (EPI) sequence with following parameters: TR = 2 s, TE = 30 ms, slice thickness = 3.3 mm, 35 slices, FOV = 220 × 220 mm^2^, and matrix = 64 × 64. 150 time points were collected for each subject. Subjects were instructed to lie still and keep their eyes open during the scan acquisition.

### Data preprocessing

Data preprocessing was performed with a standard pipeline^40^ using FSL^41^ and SPM8 (http://www.fil.ion.ucl.ac.uk/spm/). First, skull stripping was performed using FSL. SPM8-based processing steps were then performed using batch scripts provided within ASLtbx^42^. Briefly, the rsfMRI images were corrected for slice timing and the six directions of head motion (3 translations and 3 rotations). The images were subsequently smoothed with a full width half maximum (FWHM) 6 mm Gaussian kernel and band-pass filtered (0.009–0.1 Hz). CSF and white matter signals, as well as the motion parameters and their derivatives, were included in the model as covariates of no interest.

Each subject’s BEN map was calculated from the preprocessed rsfMRI images using the BENtbx (https://cfn.upenn.edu/~zewang/software.html)^20^. Sample entropy was used as the approximate entropy metric^43^. Sample entropy quantifies the temporal irregularity (incoherence) of a time series through calculating the “logarithmic likelihood” that a small segment (with a window length equal to *‘m’*) of the data matches with other segments will still match the others if the segment length increases by 1. “match” is defined by a cut-off threshold *‘r’*., Based on our previous work^20^, the window length was set to 3 and the cut off threshold was set to 0.6. Denoting the rsfMRI data of one voxel by *x* = [*x*_1_, *x*_2_, …, *x*_*N*_], where N is the number of acquisitions, SampEn calculation starts with forming a series of vector sequences, each with m consecutive points extracted from *x*: *u*_*i*_ = [*x*_*i*_, *x*_*i*+1_, …, *x*_*i*+*m* − 1_], where i = 1…(*N*−*m* + 1), and *m* is the pre-defined window size (3, as stated above). For the purpose of description, we named these vectors as embedded vectors. Using a pre-specified distance threshold *r* (0.6), the number of embedded vectors *u*_*j*_ (*J* = 1*…*(*N*−*m*)*, and J*≠*i*) whose distance from *u*_*i*_are less than *r* is recorded by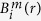. The same procedure is repeated for the dimension of *m* + 1 to get 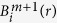. Then by averaging across all possible vectors, we have


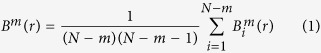



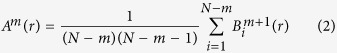


And SampEn is calculated as:


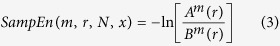


Each subject’s BEN map was first calculated in the native rsfMRI image space, then nonlinearly warped to the Montreal Neurological Institute (MNI) standard space using SPM8 and resampled with a resolution of 2 × 2 × 2 mm^3^. The name of regions of significance were identified using the Automated Anatomical Labeling (AAL) atlas^44^.

Since BEN is still new in fMRI, we also conducted a standard FC analysis. Seed-based FCs were calculated using the 5 mm-radius sphere seeds published by Power *et al*.^45^. We chose several seeds located in dACC, posterior cingulate cortex (PCC) and bilateral anterior insula. For each seed, the mean time signal was extracted and correlated with the time signal of all voxels across the brain. The z scores of the correlation maps were calculated and used for group analysis.

### Group level analysis

The following group level analyses were performed. 1) The group level BEN difference between the smokers and the controls was assessed with the massive univariate two-sample t-test implemented in SPM8 using the spatially normalized BEN maps. Only grey matter voxels were considered in this study by using a grey matter mask (created from the grey matter priors provided in SPM8, thresholded with probability value >0.4). The significance level was set to be p < 0.05 (FWE corrected). A cluster threshold of ≥100 voxels was used to remove sporadic voxels. 2) In both the smoker group and the control group, BEN differences between female subjects and male subjects were assessed using the same statistical analysis step as mentioned above. 3) For smokers, we examined the correlation between BEN and each of the smoking behavior scores (i.e. CPD, years-smoking, pack-years, and FTND) using the multiple linear regression model provided in SPM8. Each smoking behavior measure such as years-smoking was entered as the covariate of interest. Age and sex were included as the nuisance variables. The multiple regression was performed for each voxel of the entire brain. A significance level of p < 0.005 (un-corrected) was used in combination with a cluster-wise threshold of cluster extent ≥ 100 voxels. 4) Smokers were divided into two subgroups according to sex and the above BEN vs smoking behavior correlation analysis was performed for each subgroup independently. 5) Group level FC difference between the smokers and controls was assessed using two-sample t-test. Grey matter mask was used and the result images were thresholded with p < 0.005 (un-corrected) and cluster extent of ≥ 100 voxels.

## Results

### BEN results in smokers versus controls

**Figure 1:**
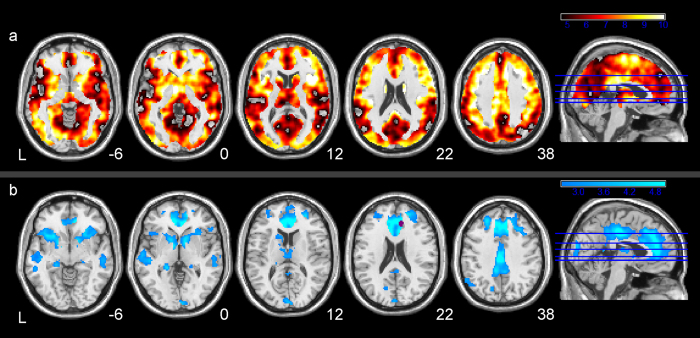
Resting BEN and functional connectivity differences between smokers and controls. (**a**) BEN difference between smokers and controls. Hot color reflects greater BEN in smokers. The map was thresholded at a voxel-wise threshold of p < 0.05 (FWE corrected) and cluster size > 100 voxels. (**b**) dACC functional connectivity difference between smokers and controls. Violet color shows the seed region. Cool color reflects smaller functional connectivity in smokers. The map was thresholded at a voxel-wise threshold of p < 0.005 (un-corrected) and cluster size > 100 voxels. L = the left side of the brain. The digital numbers to the right of each axial image and the blue lines in the sagittal image indicate the physical locations along z direction (mm) of the corresponding axial images in MNI space.

Figure 1 shows the smoker versus (vs) control contrast analysis results. Smokers showed higher BEN than controls throughout many regions in the cortex, with the greatest BEN increases located in lateral striatum (including caudate and putamen), prefrontal cortex, insula, middle and superior frontal cortex, and visual cortex (Fig. 1a, Supplementary Table S1).

Reduced dACC FC was identified in smokers than controls in insula, striatum and several default-mode-network regions (e.g. ACC, PCC, mPFC, temporal cortex and hippocampus) (Fig. 1b). Strongly reduced PCC FC, left anterior insula FC and right anterior insula FC were also identified and were presented in Supplementary Fig. S1.

### Correlations between BEN and smoking behavioral measures

**Figure 2:**
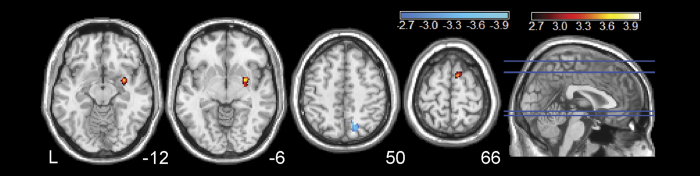
Correlation between BEN and years-smoking in smokers. Hot/cool color means positive/negative correlation, respectively. Correlation results were thresholded at p < 0.005 (un-corrected) and cluster size >100 voxels. L = the left side of the brain. The digital numbers to the right of each axial image and the blue lines in the sagittal image indicate the physical locations along z direction (mm) of the corresponding axial images in MNI space.

For the smoker group, the correlation between BEN and years-smoking is presented in Fig. 2 and Supplementary Table S2. A positive correlation between BEN and years-smoking was observed in the right putamen/amygdala/pallidum, the supplementary motor area and the prefrontal cortex. A negative correlation between BEN and years-smoking was found in the right superior parietal/precuneus.

With the statistical threshold used in this article (p < 0.005, un-corrected, and cluster extent ≥ 100), no significant correlations were identified between BEN and CPD, pack-years, or FTND in the smoker group.

### Sex effects in resting BEN

**Figure 3:**
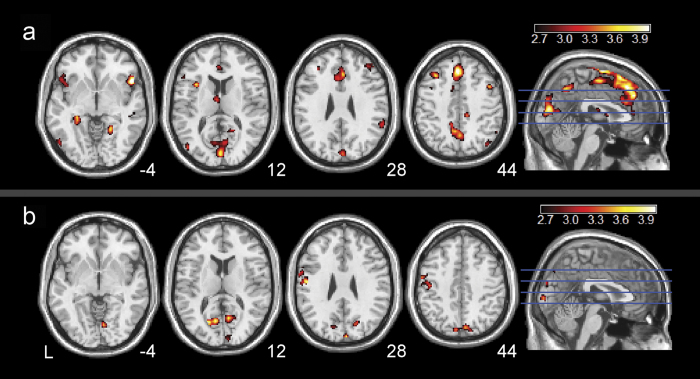
Gender effects on resting BEN. Both (**a**) normal controls and (**b**) chronic smokers showed greater BEN in female subjects than male subjects (shown in hot color). The statistical parametric maps (two sample t-maps) were thresholded at p < 0.005 (uncorrected) and cluster size > 100 voxels. Hot color means higher brain entropy in the females. L means left side of the brain. L = the left side of the brain. The digital numbers to the right of each axial image and the blue lines in the sagittal image indicate the physical locations along z direction (mm) of the corresponding axial images in MNI space.

As shown in Fig. 3 and Supplementary Tables S3–S4, female subjects showed higher resting BEN than their male counterparts in both control group (Fig. 3a) and smoker group (Fig. 3b)). Compared to male controls, female controls showed higher resting BEN in both medial and lateral parts of the prefrontal cortex, the lateral orbitofrontal cortex (OFC), bilateral insula, ACC, thalamus, precuneus, and visual cortex (Fig. 3a, Supplementary Table S3). Smokers had less widespread resting BEN difference between females and males (Fig. 3b, Supplementary Table S4), with female smokers showing higher BEN in left primary motor cortex, precuneus, and visual cortex.

### Sex differences in correlations between BEN and smoking behavioral measures

**Figure 4:**
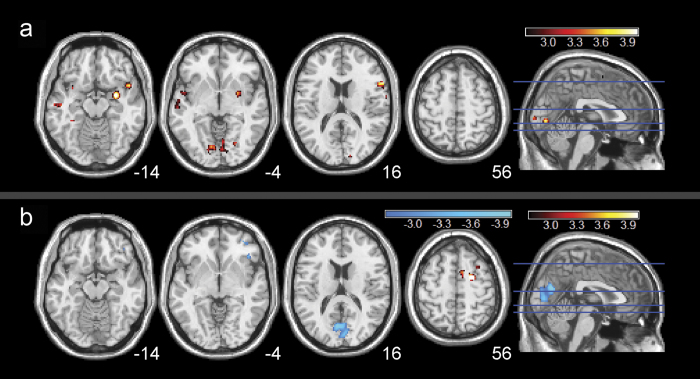
Correlations between resting BEN and years-smoking in both female and male smokers. Both (**a**) female smokers and (**b**) male smokers showed significant correlation with years-smoking. Statistical results were thresholded at p < 0.005 (un-corrected) and cluster size > 100 voxels. Hot/cool color means positive/negative correlation, respectively. L = the left side of the brain. The digital numbers to the right of each axial image and the blue lines in the sagittal image indicate the physical locations along z direction (mm) of the corresponding axial images in MNI space.

Different patterns emerged between male and female smokers in associations with smoking behavioral measures. Female smokers showed a positive correlation between BEN and years-smoking in several regions including bilateral inferior frontal cortex, right amygdala, bilateral calcarine, bilateral lingual, right putamen, and left middle/superior temporal cortex (Fig. 4a, Supplementary Table S5). Male smokers showed a positive correlation between BEN and years-smoking in right dorsolateral prefrontal cortex, and a negative correlation in visual cortex (Fig. 4b, Supplementary Table S6). (In female smokers, after loosening the extent threshold to 30 voxels, we observed a negative correlation between BEN and years-smoking in a small cluster (38 voxels) in the right parietal/precuneus regions).

[Table t1]The correlations between BEN and FTND, CPD and pack-years in different sex subgroups were presented in the supplemental figures (Supplementary Figs S2–S4). Female smokers showed significant negative correlation between BEN and FTND in right middle and inferior occipital cortex, while male smokers presented positive correlation in precuneus region and negative correlation in bilateral postcentral cortex and middle and inferior frontal cortex (Supplementary Fig. S2). Female smokers showed negative correlation between CPD and BEN in left temporal cortex and insula, while male smokers presented positive correlation in right temporal pole, left precentral cortex and left frontal cortex, and negative correlation in left post central cortex (Supplementary Fig. S3). Female smokers showed positive correlation between pack-years and BEN in right middle and inferior occipital lobe, while male smokers presented positive correlation in right middle temporal cortex and negative correlation in right middle occipital lobe (Supplementary Fig. S4).

## Discussion

The present study assessed the regional functional brain alterations in satiated chronic smokers using resting BEN mapping. As compared to controls, smokers showed globally increased BEN, demonstrating more irregularly fluctuating resting brain activity. This global BEN increase might reflect the global brain activity alterations due to chronic cigarette smoking. Nicotine stimulates the brain by binding to endogenous nicotinic acetylcholine receptors (nAchRs)^46^. Through its activation of nAchRs nicotine also indirectly activates dopaminergic and glutamatergic receptors and systems^47^,^48^. Thus, nicotine has the capacity to alter numerous brain systems including those involved in reward, mood, compulsivity, impulsivity, and motivation (limbic), and those involved in attention, inhibition, and cognitive control (prefrontal cortex)^1^,^46^,^49^,^50^,^51^. Repetitive chronic cigarette smoking is likely to disrupt functional dynamics within the brain, which can be subsequently reflected by greater entropy in the resting brain.

Higher entropy means more irregularity and more high-frequency fluctuations. Technically, having more high-frequency and irregular fluctuations in correlated signals reduces their correlation. In our additional FC analysis, the smokers showed strongly reduced dACC FC, PCC FC and bilateral anterior insula FC than the controls, which was in accordance with previous literature^8^,^10^,^11^. Our findings of strongly reduced FC, together with the findings of previously literature, partially support our findings of higher BEN in the smokers.

The globally increased BEN is unlikely induced by vascular effects of nicotine. Our imaging data were acquired 20–25 minutes after cigarette smoking, and the resting state fMRI was acquired about 10 minutes after the start of the scan session. This 30–35 minutes post-smoking period should be enough to avoid the vascular effects of nicotine^39^.

Our result showed relatively higher BEN increase in prefrontal cortex, lateral striatum, visual cortex, and motor area, which is consistent with the fact that nicotine dependence has the strongest impact on attention, decision making, reward, and action/motion function that are subserved by those brain regions. This postulation is further supported by our correlation analysis between BEN and smoking behavior measures, where we found that higher BEN in the mesocortico limbic area, prefrontal cortex, and supplementary motor area (SMA) predicts more years-smoking. Since years-smoking is associated with the level of nicotine dependence (FTND) and pack-years, these correlations suggest a beneficial effect of nicotine on the resting functional dynamics in regions associated with those functions. Resting brain activity in the default mode network has been postulated to consolidate normal brain functions^52^. We found that longer years-smoking was related with lower BEN in default mode network, which may indicate that more organized spontaneous brain activity is required to cope with the functional disruptions caused by more severe nicotine dependence. This possibility is supported by previous functional connectivity (FC) studies^53^,^54^, where reduced FC was found in the precuneus and other default mode network areas in cigarette smokers.

Sex differences of BEN existed in both controls and smokers. Female controls showed higher BEN than male controls in bilateral OFC, thalamus, insula, ACC, bilateral DLPFC, parietal cortex and precuneus, indicating greater irregular resting brain activity within those regions in female controls. The findings of higher irregularity in default mode network regions (OFC, parietal cortex, and precuneus) in female controls than male controls are in accordance with previous large cohort study of healthy controls^40^, in which these regions were found to have lower FC, lower regional rsfMRI data coherence, and reduced resting state network activity in the females. Increased BEN in the limbic system regions (OFC, thalamus, ACC and insula) may indicate that female controls are more irregular in terms of limbic brain functions such as reward, motivation, sensorimotor function. These regional BEN sex differences in the control group might be contributed by the hormonal effects^40^. Interestingly, smokers showed spatially less distributed BEN sex differences than controls. Additional analysis found that the smoker-control BEN difference was more prominent in males than in females (Supplementary Fig. S5). These results suggest that male smokers had larger nicotine dependence-related resting BEN changes than female smokers. One reason for this diminishing sex BEN difference in smokers could be the sex difference of nicotine dependence^34^. In the regression analysis, BEN in female smokers accounted for more positive BEN vs years-smoking correlation found in the entire group regression analysis, while male smokers showed positive BEN vs years-smoking only in the right DLPFC. Together, these findings suggest that nicotine dependence had bigger effects on the resting BEN in males than in females especially in brain regions that are associated with reward, motivation, and sensorimotor functions which are known to be affected by cigarette smoking^46^.

The nictoine withdrawal status may have different effects on the resting state in smokers^55^,^56^,^57^. In this study, the recruited smokers were minimally nicotine deprived, which may explain the seemly opposition of our sex difference findings to what was reported recently^58^, where smoking abstinent females showed greater connectivity than men within the default mode network.

In summary, we found globally increased resting BEN in chronic smokers, which was related to years-smoking in the right limbic area and frontal region. We also found sex effects of resting BEN in non-smokers as well as in smokers, however the BEN difference between male and female smokers was markedly reduced than non-smokers. The altered resting BEN entropy patterns and the patterns related to smoking behavior provided new aspects of smoking-related brain alterations, helping to better understand the underlying neural mechanisms of nicotine dependence from a temporal dynamics perspective. This is different from the existing nicotine dependence resting state studies wherein the information gleaned reflects static functional connectivity or resting state network activity, i.e. the correlation between time series of different regions. As an alternative and additive method, BEN provides a method to characterize the temporal dynamics of the resting brain. The BEN mapping technique and our findings might provide new hints for developing effective approaches to treat cigarette dependence by targeting the irregularities of the fluctuating activity within the resting brain.

## Additional Information

**How to cite this article**: Li, Z. *et al*. Hyper-resting brain entropy within chronic smokers and its moderation by Sex. *Sci. Rep.* **6**, 29435; doi: 10.1038/srep29435 (2016).

## Supplementary Material

Supplementary Information

## Figures and Tables

**Table 1 t1:** Demographic of study sample.

	Smokers (N = 68)	Controls (N = 66)	Smokers vs. Controls Statistics	
	Mean(s.d.)	Mean(s.d.)	T	p
Age, years	35.3 (12.6)^a^	33.0 (8.2)^b^	1.26	0.21
FTND	4.7 (1.6)			
CPD	14.5 (6.1)			
Years-smoking	15.4 (11.5)			
Pack-years	11.4 (9.8)			
	N (%)	N (%)	χ^2^	p
Male	35 (51.5%)	33 (50.0%)	0.03	0.87
Ethnicity^c^			3.99	0.26
Caucasian	34 (50.0%)	19 (34.5%)		
African American	27 (39.7%)	25 (45.5%)		
Asian	5 (7.4%)	7 (12.7%)		
Others	2 (2.9%)	4 (7.3%)		

Abbreviations: s.d., standard deviation; FTND: the Fagerstrom Test for Nicotine Dependence; CPD, the self-reported quantities of cigarettes per day; years-smoking, the duration of smoking history, it was calculated by subtracting the age at assessment with the age at smoking initiation; Pack-years, summarization of the amount of cigarettes smoked over a lifetime, 1 pack-years is equal to smoking a pack (20 manufactured cigarettes) per day for a year, it was calculated as CPD/20 *years-smoking; a, age ranged 19–58 for smokers; b, age ranged 21–51 for controls; c, based on available ethnicity information of 68 smokers and 55 controls.
