# Supplementary material for: Hyper-resting brain entropy within chronic smokers and its moderation by Sex
Source: Sci Rep. 2016 Jul 5;6:29435. doi: 10.1038/srep29435 (PMC4932513; doi:10.1038/srep29435)
Supplement: Supplementary Information [file srep29435-s1.doc]

Hyper-resting brain entropy within chronic smokers and its moderation by Sex

Zhengjun Li^1^, Zhuo Fang^1, 2, 3^, Nathan Hager^1^, Hengyi Rao^2, 4^, Ze Wang^5, 6, 7, 1, *^

^1^Department of Psychiatry, Perelman School of Medicine, University of Pennsylvania, Philadelphia, Pennsylvania, USA, ^2^Laboratory of Applied Brain and Cognitive Sciences, College of International Business, Shanghai International Studies University, Shanghai, China, ^3^The Brain and Mind Institute, Department of Psychology, Western University, London, Ontario, Canada, ^4^Department of Neurology, Perelman School of Medicine, University of Pennsylvania, Philadelphia, Pennsylvania, USA, ^5^Center for Cognition and Brain Disorders, Institutes of Neurological Science, Hangzhou Normal University, ^6^Affiliated Hospital of Hangzhou Normal University, ^7^Zhejiang Key Laboratory for Research in Assessment of Cognitive Impairments, Hangzhou, Zhejiang Province, China

**Supplementary Figures (Female–male separated correlations with behavior scores and two sample t test between smokers and controls)**


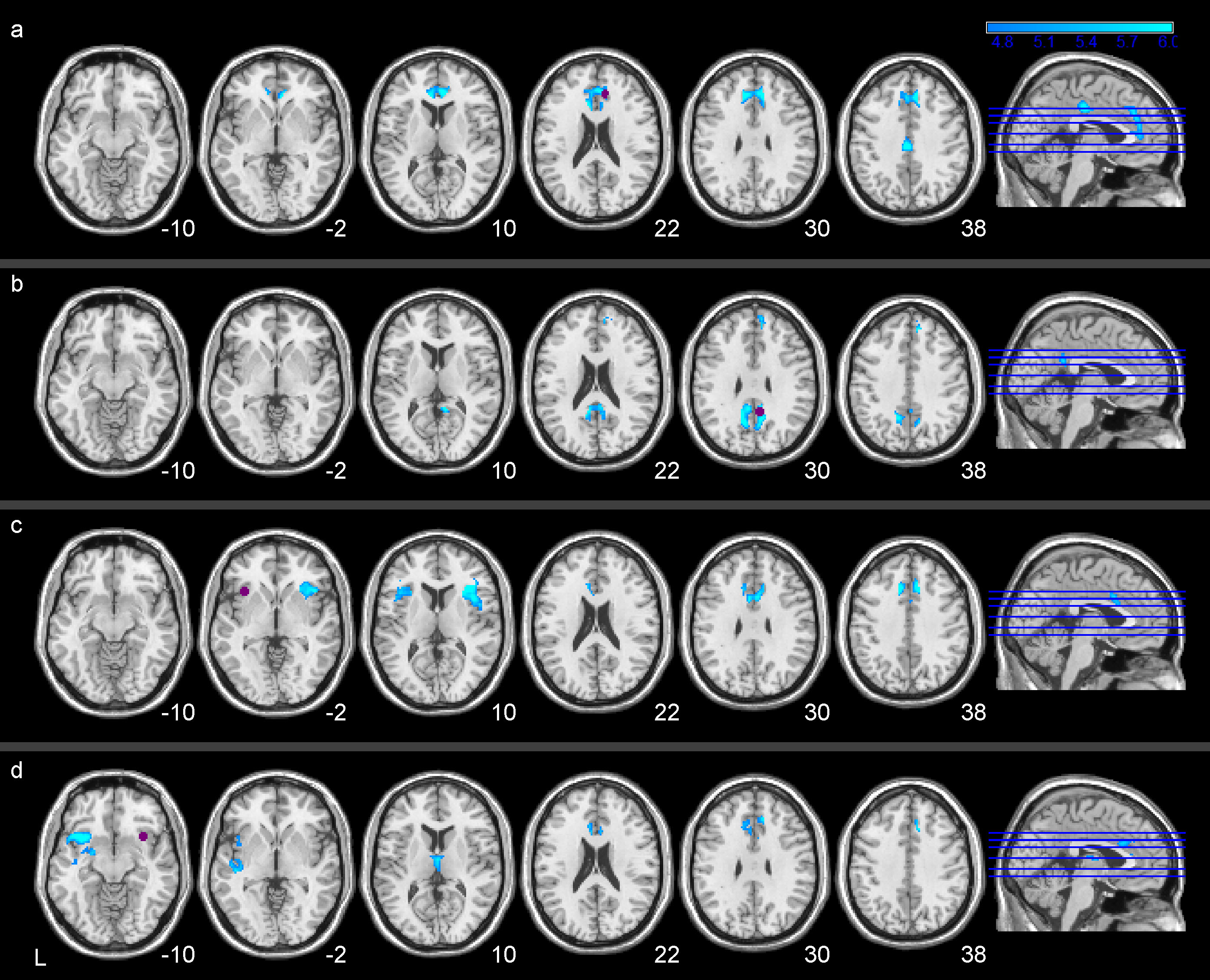


Figure S1. Resting functional connectivity difference between smokers and controls. a), dorsal anterior cingulate cortex (dACC) functional connectivity difference between smokers and controls. b), posterior cingulate cortex (PCC) functional connectivity difference between smokers and controls. c), left anterior insular functional connectivity difference between smokers and controls. d), right anterior insular functional connectivity difference between smokers and controls. Violet color shows the seed regions. Cool color reflects smaller functional connectivity in smokers. The maps were thresholded at a voxel-wise threshold of p < 0.05 (FWE corrected) and cluster size > 100 voxels. L = the left side of the brain. The digital numbers to the right of each axial image and the blue lines in the sagittal image indicate the physical locations along z direction (mm) of the corresponding axial images in MNI space. (Please note that Fig. S1a is the more stringently thresholded version of Fig. 1b of the main manuscript.)

*
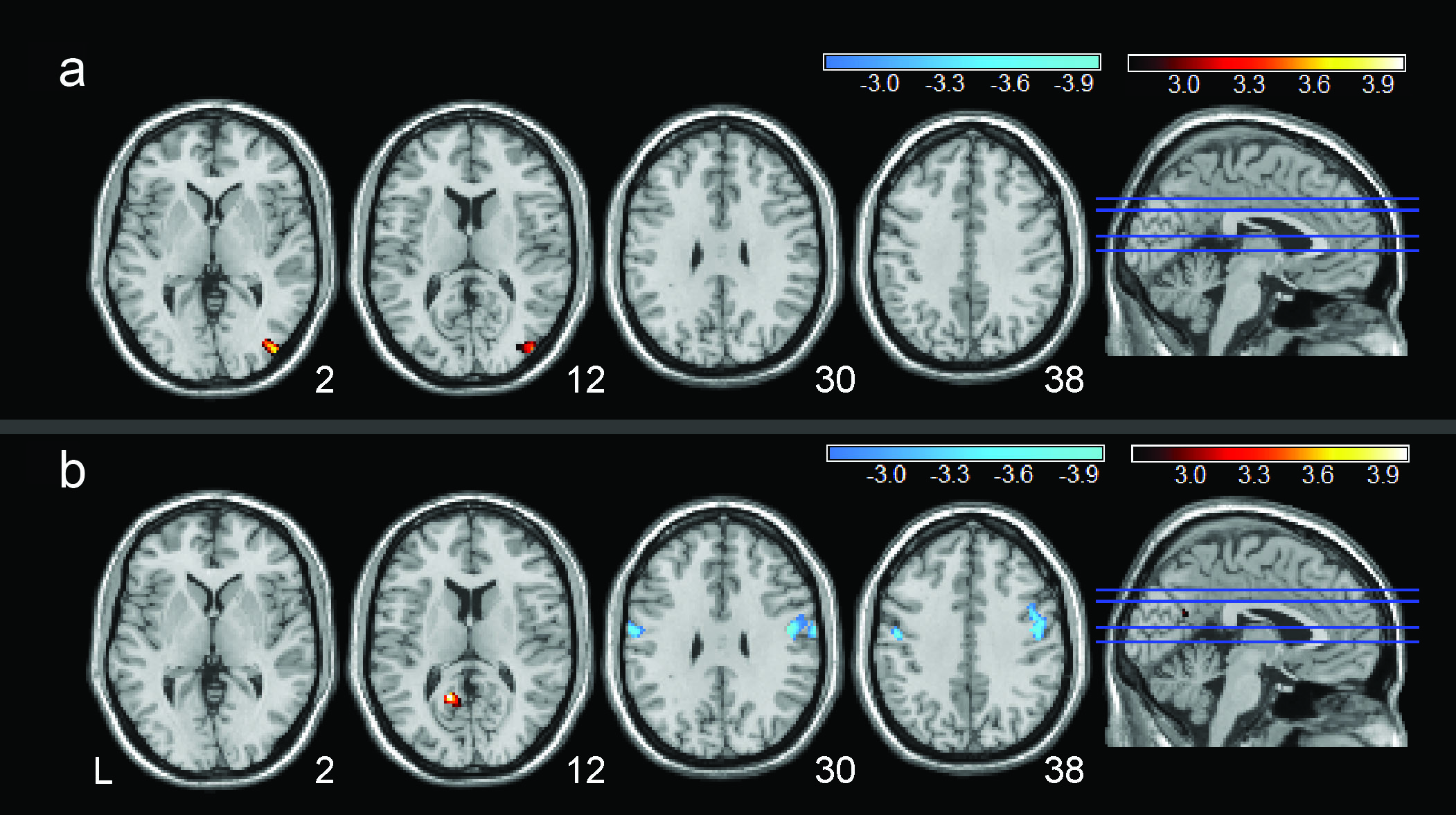
Figure S2. Correlations between resting BEN and FTND in A) female smokers, B) male smokers. Statistical results were thresholded at p < 0.005 (un-corrected) and cluster size >100 voxels. Hot/cool color means positive/negative correlation, respectively. L = the left side of the brain. The digital numbers to the right of each axial image and the blue lines in the sagittal image indicate the physical locations along z direction (mm) of the corresponding axial images in MNI space.*


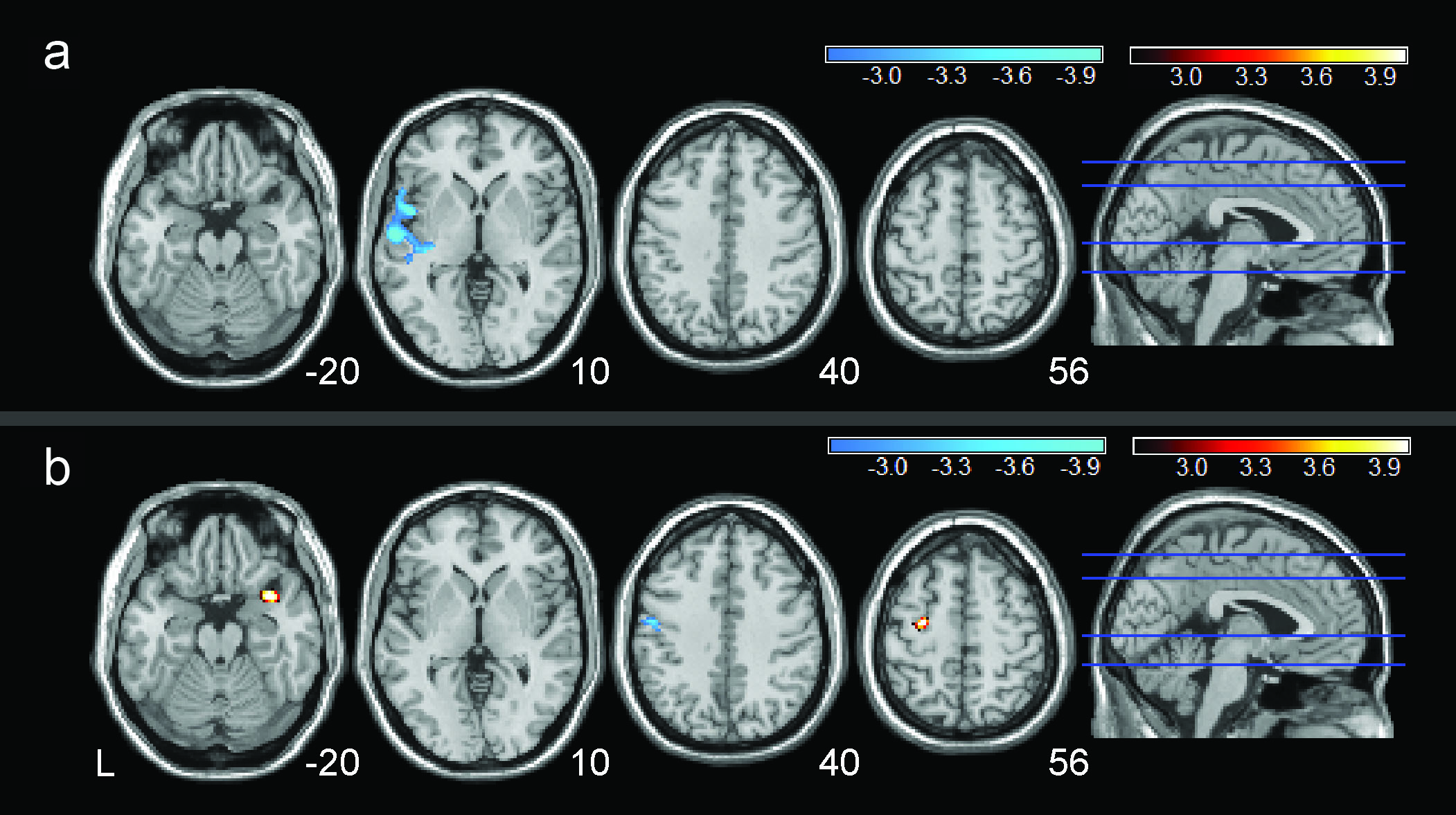


Figure S3. Correlations between resting BEN and CPD in a) female smokers, and in b) male smokers. Statistical results were thresholded at p < 0.005 (un-corrected) and cluster size >100 voxels. Hot/cool color means positive/negative correlation, respectively. L = the left side of the brain. The digital numbers to the right of each axial image and the blue lines in the sagittal image indicate the physical locations along z direction (mm) of the corresponding axial images in MNI space.


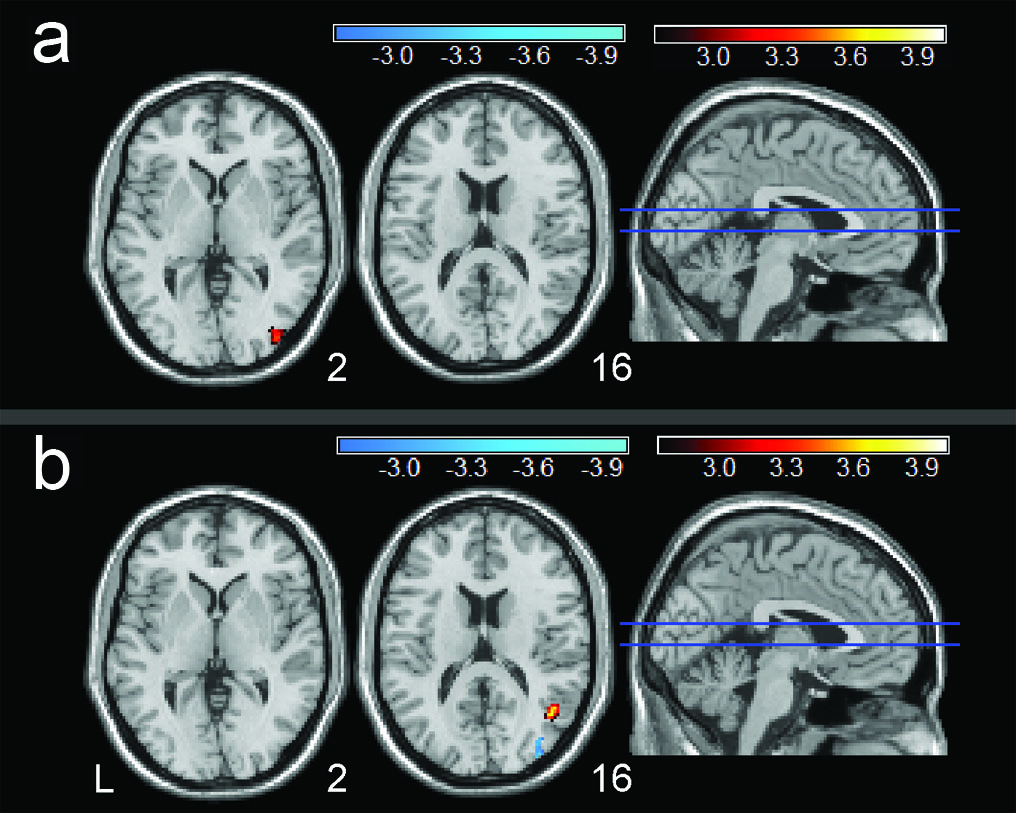


Figure S4. Correlations between resting BEN and pack-years in a) female smokers, and in b) male smokers. Statistical results were thresholded at p < 0.005 (un-corrected) and cluster size >100 voxels. Hot/cool color means positive/negative correlation, respectively. L = the left side of the brain. The digital numbers to the right of each axial image and the blue lines in the sagittal image indicate the physical locations along z direction (mm) of the corresponding axial images in MNI space.


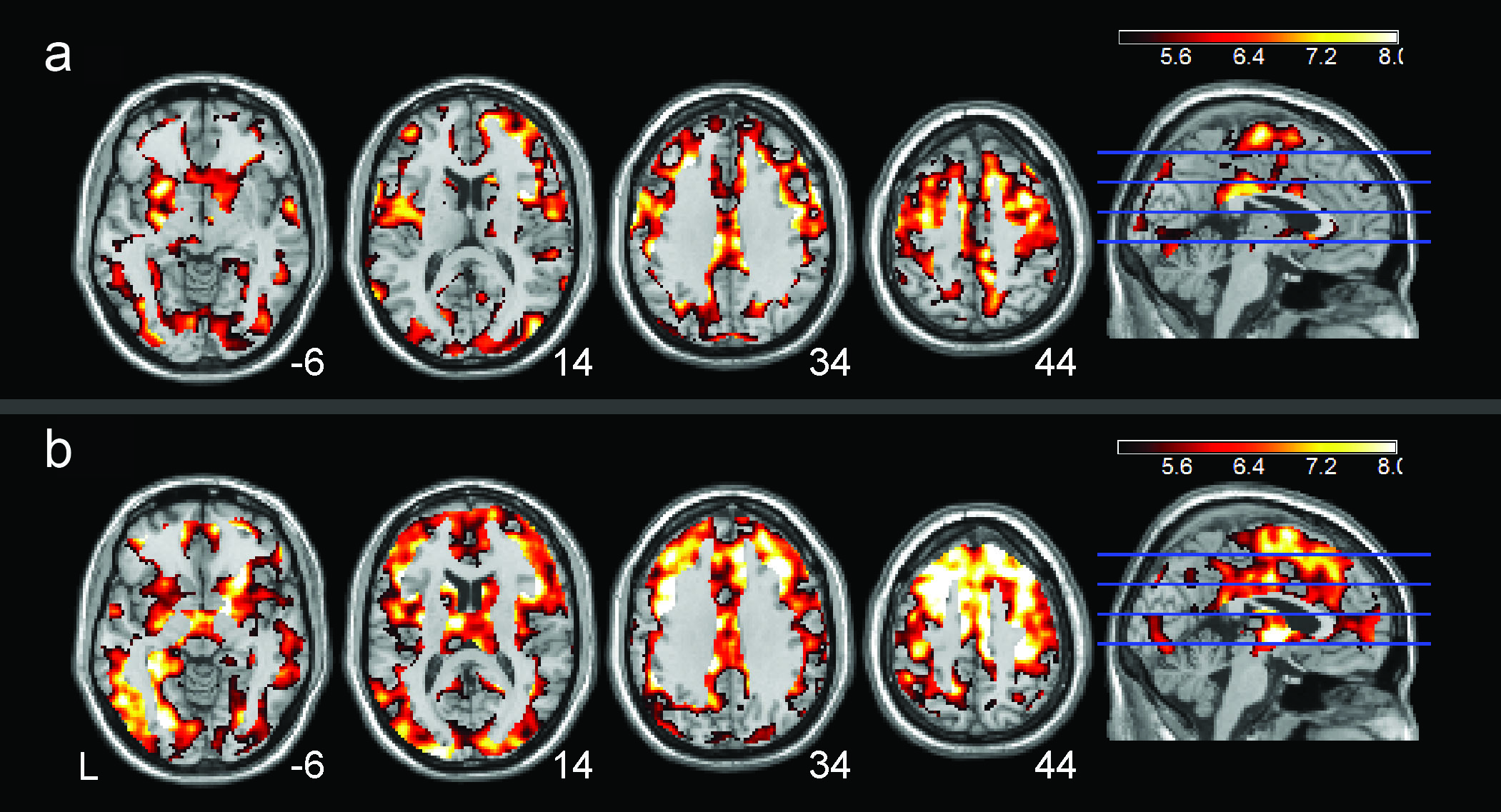


Figure S5. Resting BEN difference in smokers versus controls in both females and males. a) BEN difference between female smokers and female controls, b) BEN difference between male smokers and male controls. Hot color reflects greater BEN in smokers. The map was thresholded at a voxel-wise threshold of p < 0.05 (FWE corrected) and cluster size > 100 voxels. L = the left side of the brain. The digital numbers to the right of each axial image and the blue lines in the sagittal image indicate the physical locations along z direction (mm) of the corresponding axial images in MNI space.

**Supplementary Tables (Peak coordinates of main manuscript figures)**

Table S1. Peak regions of the smoker versus control BEN analysis. Only representative locations identified using a superior threshold of p < 10^-10^ (FWE corrected, t > 9.0) are presented.

| **Cluster** | **Size(Vx)** | **Peak Region** | **t value** | **MNI Coordinates (mm)** | | |
| --- | --- | --- | --- | --- | --- | --- |
|  |  |  |  | **x** | **y** | **z** |
| 1 | 14053 | R. Inferior Frontal Operculum | 14.09 | 38 | 10 | 14 |
|  |  | R. Middle Frontal Gyrus | 12.94 | 34 | 4 | 54 |
|  |  | L. Supplementary Motor Area | 12.61 | -12 | 2 | 64 |
|  |  | R. Superior Frontal Gyrus | 12.46 | 18 | 34 | 42 |
|  |  | R. Superior Frontal Gyrus - Medial Part | 12.43 | 10 | 28 | 40 |
|  |  | L. Precentral Gyrus | 12.38 | -20 | -16 | 62 |
|  |  | R. Precentral Gyrus | 12.3 | 64 | 8 | 24 |
|  |  | L. Middle Frontal Gyrus | 12.1 | -28 | 10 | 50 |
|  |  | L. Superior Frontal Gyrus | 12.04 | -26 | 28 | 34 |
|  |  | R. Supplementary Motor Area | 12.03 | 10 | -14 | 66 |
|  |  | R. Paracentral Lobule | 11.99 | 10 | -32 | 56 |
|  |  | R. Posterior Cingulate Cortex | 11.92 | 8 | -40 | 26 |
|  |  | R. Inferior Frontal Gyrus - pars triangulars | 11.91 | 50 | 38 | 26 |
|  |  | R. Postcentral Gyrus | 11.79 | 30 | -30 | 54 |
|  |  | L. Inferior Frontal Operculum | 11.71 | -36 | 18 | 32 |
|  |  | R. Middle Cingulate Cortex | 11.61 | 12 | 16 | 38 |
|  |  | L. Inferior Parietal Lobule | 11.4 | -42 | -28 | 38 |
|  |  | L. Middle Cingulate Cortex | 11.26 | -6 | -10 | 34 |
|  |  | L. Precuneus | 11.23 | -12 | -52 | 44 |
|  |  | L. Insula | 11.22 | -30 | 20 | 12 |
|  |  | L. Posterior Cingulate Cortex | 11.22 | -8 | -36 | 32 |
|  |  | L. Thalamus | 11.1 | -6 | -4 | 0 |
|  |  | L. Putamen | 11.09 | -28 | -10 | -8 |
|  |  | R. Pallidum | 10.94 | 14 | 4 | -4 |
|  |  | L. Rolandic Operculum | 10.47 | -46 | -6 | 16 |
|  |  | R. Inferior Frontal Gyrus - Orbital Part | 10.41 | 28 | 28 | -10 |
|  |  | L. Postcentral Gyrus | 10.36 | -36 | -30 | 44 |
|  |  | L. Pallidum | 10.15 | -18 | 0 | -2 |
|  |  | R. Putamen | 10.14 | 24 | 18 | 4 |
|  |  | R. Insula | 9.93 | 30 | 30 | 0 |
|  |  | R. Anterior Cingulate Cortex | 9.86 | 12 | 30 | 26 |
|  |  | R. SupraMarginal Gyrus | 9.76 | 34 | -36 | 40 |
|  |  | L. SupraMarginal Gyrus | 9.53 | -52 | -32 | 32 |
|  |  | L. Superior Frontal Gyrus - Medial Part | 9.37 | -10 | 24 | 38 |
|  |  | L. Hippocampus | 9.26 | -30 | -12 | -16 |
|  |  | L. Cuneus | 9.23 | -18 | -62 | 26 |
|  |  | L. Inferior Frontal Gyrus - pars triangulars | 9.07 | -40 | 28 | 16 |
| 2 | 201 | L. SupraMarginal Gyrus | 11.22 | -48 | -48 | 30 |
|  |  | L. Inferior Parietal Lobule | 10.75 | -44 | -50 | 38 |
|  |  | L. Middle Occipital Gyrus | 10.36 | -30 | -62 | 36 |
|  |  | L. Angular Gyrus | 10.32 | -36 | -56 | 36 |
|  |  | L. Superior Parietal Lobule | 9.94 | -26 | -58 | 44 |
| 3 | 146 | L. Anterior Cingulate Cortex | 11.07 | -12 | 50 | 0 |
| 4 | 109 | R. Caudate | 10.74 | 14 | -4 | 22 |
|  |  | R. Thalamus | 9.73 | 16 | -16 | 18 |
| 5 | 965 | L. Inferior Occipital Gyrus | 10.68 | -30 | -78 | -10 |
|  |  | L. Inferior Temporal Gyrus | 10.54 | -44 | -42 | -16 |
|  |  | L. Fusiform Gyrus | 10.24 | -28 | -70 | -6 |
|  |  | L. Lingual Gyrus | 10 | -28 | -46 | -2 |
|  |  | L. Hippocampus | 9.96 | -34 | -38 | -6 |
|  |  | L. Parahippocampal Gyrus | 9.15 | -28 | -36 | -14 |
| 6 | 100 | L. Middle Frontal Gyrus | 10.2 | -32 | 50 | 0 |
|  |  | L. Inferior Frontal Gyrus - pars triangulars | 9.89 | -40 | 38 | 4 |
| 7 | 115 | R. Cuneus | 10.01 | 16 | -90 | 8 |
|  |  | R. Superior Occipital Gyrus | 9.15 | 14 | -96 | 22 |

Abbreviations: L, left; R, right.

Table S2. Peak regions of the correlation between BEN and years-smoking.

| **Cluster** | **Size(Vx)** | **Peak Region** | **t value** | **MNI Coordinates (mm)** | | |
| --- | --- | --- | --- | --- | --- | --- |
|  |  |  |  | **x** | **y** | **z** |
| 1 | 124 | R. Putamen | 3.84 | 30 | 2 | -8 |
| 2 | 108 | R. Supplementary Motor Area | 3.69 | 12 | 8 | 68 |
|  |  | R. Superior Frontal Gyrus | 2.9 | 18 | 8 | 62 |
|  |  |  |  |  |  |  |
| 3 | 138 | R. Precuneus | -3.82 | 16 | -68 | 48 |

Abbreviation: R, right. Significance level was defined by p < 0.005 (un-corrected) and cluster size >100 voxels.

Table S3. Peak regions of BEN gender effects (female > male) in controls.

| **Cluster** | **Size(Vx)** | **Peak Region** | **t value** | **MNI Coordinates (mm)** | | |
| --- | --- | --- | --- | --- | --- | --- |
|  |  |  |  | **x** | **y** | **z** |
| 1 | 301 | R. Inferior Frontal Operculum | 4.86 | 54 | 20 | -2 |
| 2 | 3550 | L. Supplementary Motor Area | 4.62 | -12 | -4 | 74 |
|  |  | R. Supplementary Motor Area | 4.6 | 6 | -4 | 74 |
|  |  | L. Superior Frontal Gyrus - Medial Part | 4.35 | 0 | 28 | 48 |
|  |  | L. Superior Frontal Gyrus | 3.88 | -22 | 16 | 56 |
|  |  | R. Anterior Cingulate Cortex | 3.72 | 4 | 28 | 26 |
|  |  | R. Middle Cingulate Cortex | 3.44 | 12 | -30 | 46 |
|  |  | L. Anterior Cingulate Cortex | 3.35 | -6 | 24 | 26 |
|  |  | L. Paracentral Lobule | 3.3 | -2 | -16 | 76 |
|  |  | L. Middle Cingulate Cortex | 3.16 | -10 | 4 | 40 |
|  |  | L. Middle Frontal Gyrus | 2.98 | -36 | 12 | 54 |
|  |  | L. Precentral Gyrus | 2.96 | -38 | 10 | 42 |
|  |  | R. Paracentral Lobule | 2.86 | 2 | -26 | 76 |
|  |  | R. Superior Frontal Gyrus - Medial Part | 2.83 | 10 | 38 | 52 |
|  |  | L. Precuneus | 2.83 | -4 | -42 | 72 |
| 3 | 229 | L. Thalamus | 4.17 | -10 | -18 | 18 |
|  |  | R. Caudate | 3.2 | 4 | 2 | 8 |
| 4 | 214 | R. Lingual Gyrus | 4.13 | 22 | -50 | 0 |
|  |  | R. Calcarine Sulcus | 3.42 | 20 | -50 | 10 |
| 5 | 212 | R. SupraMarginal Gyrus | 4.04 | 60 | -46 | 32 |
|  |  | R. Superior Temporal Gyrus | 3.21 | 56 | -42 | 20 |
|  |  | R. Inferior Parietal Lobule | 2.92 | 56 | -56 | 46 |
| 6 | 489 | L. Precuneus | 4.02 | -8 | -52 | 48 |
|  |  | R. Precuneus | 3.67 | 4 | -60 | 46 |
| 7 | 481 | L. Insula | 3.93 | -32 | 14 | 10 |
|  |  | L. Inferior Frontal Gyrus - pars triangulars | 3.74 | -50 | 24 | 0 |
|  |  | L. Superior Temporal Pole | 3.39 | -42 | 24 | -18 |
|  |  | L. Inferior Frontal Gyrus - Orbital Part | 3.06 | -46 | 22 | -10 |
| 8 | 640 | L. Cuneus | 3.92 | 2 | -84 | 16 |
|  |  | R. Calcarine Sulcus | 3.18 | 8 | -70 | 12 |
|  |  | L. Calcarine Sulcus | 3.09 | -2 | -70 | 14 |
| 9 | 144 | R. Middle Frontal Gyrus | 3.74 | 46 | 12 | 48 |
| 10 | 148 | R. Angular Gyrus | 3.69 | 50 | -72 | 38 |
| 11 | 281 | L. Middle Frontal Gyrus | 3.67 | -32 | 28 | 44 |
| 12 | 106 | L. Cerebellum Crus I | 3.66 | -42 | -64 | -22 |
|  |  | L. Fusiform Gyrus | 2.88 | -40 | -52 | -14 |
| 13 | 169 | L. Hippocampus | 3.63 | -26 | -36 | -2 |
| 14 | 139 | L. Angular Gyrus | 3.56 | -48 | -64 | 36 |
|  |  | L. Inferior Parietal Lobule | 3.15 | -52 | -54 | 38 |
| 15 | 177 | L. Middle Occipital Gyrus | 3.3 | -46 | -76 | 12 |
|  |  | L. Middle Temporal Gyrus | 3.23 | -52 | -66 | -2 |
| 16 | 102 | R. Middle Frontal Gyrus | 3.29 | 40 | 40 | 30 |
| 17 | 106 | R. MIddle Temporal Gyrus | 3.11 | 54 | -26 | -6 |

Abbreviation: L, left; R, right. Significance level was defined by p < 0.005 (un-corrected) and cluster size >100 voxels.

Table S4. Peak regions of BEN gender effects (female > male) in smokers.

| **Cluster** | **Size(Vx)** | **Peak Region** | **t value** | **MNI Coordinates (mm)** | | |
| --- | --- | --- | --- | --- | --- | --- |
|  |  |  |  | **x** | **y** | **z** |
| 1 | 224 | R. Postcentral Gyrus | 4.53 | 28 | -38 | 70 |
| 2 | 591 | L. Postcentral Gyrus | 4.53 | -50 | -10 | 32 |
|  |  | L. Precentral Gyrus | 3.28 | -60 | 0 | 36 |
| 3 | 667 | L. Calcarine Sulcus | 4.33 | -10 | -64 | 8 |
|  |  | R. Cuneus | 3.61 | 12 | -78 | 40 |
|  |  | L. Precuneus | 3.42 | -6 | -80 | 46 |
|  |  | L. Cuneus | 3.1 | -16 | -72 | 36 |
| 4 | 135 | L. Superior Temporal Gyrus | 4.01 | -66 | -44 | 22 |
|  |  | L. Middle Temporal Gyrus | 3.21 | -64 | -56 | 18 |
| 5 | 776 | R. Cuneus | 3.88 | 6 | -90 | 26 |
|  |  | R. Calcarine Sulcus | 3.72 | 10 | -64 | 10 |
|  |  | R. Lingual Gyrus | 3.67 | 10 | -74 | 0 |
|  |  | L. Calcarine Sulcus | 3.58 | 4 | -94 | 4 |
|  |  | R. Cerebellum Crus VI | 2.86 | 12 | -76 | -14 |
| 6 | 202 | L. Postcentral Gyrus | 3.48 | -30 | -44 | 62 |
|  |  | L. Precentral Gyrus | 3.47 | -30 | -16 | 70 |
|  |  | L. Superior Parietal Lobule | 2.85 | -18 | -48 | 70 |

Abbreviation: L, left; R, right. Significance level was defined with p < 0.005 (un-corrected) and cluster size >100 voxels.

Table S5. Peak regions of BEN vs years- smoking in female smokers.

| **Cluster** | **Size(Vx)** | **Peak Region** | **t value** | **MNI Coordinates (mm)** | | |
| --- | --- | --- | --- | --- | --- | --- |
|  |  |  |  | **x** | **y** | **z** |
| 1 | 236 | R. Amygdala | 4.73 | 32 | -2 | -12 |
| 2 | 174 | L. Middle Temporal Gyrus | 4.14 | -60 | -18 | 0 |
|  |  | L. Superior Temporal Gyrus | 3.59 | -44 | -14 | -10 |
| 3 | 452 | R. Lingual Gyrus | 4.12 | 6 | -80 | 0 |
|  |  | L. Lingual Gyrus | 3.91 | -6 | -78 | -2 |
|  |  | L. Calcarine Sulcus | 3.88 | -8 | -88 | 0 |
|  |  | R. Calcarine Sulcus | 3.51 | 14 | -86 | 8 |
|  |  | R. Cuneus | 3.35 | 14 | -88 | 18 |
| 4 | 105 | R. Inferior Frontal Operculum | 3.97 | 62 | 16 | 14 |
|  |  | R. Rolandic Operculum | 3.34 | 66 | 0 | 12 |
| 5 | 100 | L. Inferior Frontal Operculum | 3.8 | -58 | 6 | 10 |

Abbreviation: L, left; R, right. Significance level was defined with p < 0.005 (un-corrected) and cluster size >100 voxels.

Table S6. Peak regions of BEN vs years- smoking in male smokers.

| **Cluster** | **Size(Vx)** | **Peak Region** | **t value** | **MNI Coordinates (mm)** | | |
| --- | --- | --- | --- | --- | --- | --- |
|  |  |  |  | **x** | **y** | **z** |
| 1 | 132 | R. Superior Frontal Gyrus | 5.05 | 24 | -2 | 56 |
|  |  | R. Supplementary Motor Area | 3.62 | 8 | 4 | 54 |
|  |  |  |  |  |  |  |
| 2 | 698 | R. Cuneus | -4.38 | 6 | -70 | 20 |
|  |  | L. Calcarine Sulcus | -4 | -4 | -72 | 16 |
| 3 | 104 | R. Inferior Frontal Gyrus - Orbital Part | -3.39 | 44 | 28 | -4 |

Abbreviation: L, left; R, right. Significance level was defined with p < 0.005 (un-corrected) and cluster size >100 voxels.
